# Glomerular disease and pregnancy: contributions from a Brazilian cohort study

**DOI:** 10.1590/2175-8239-JBN-2025-E020en

**Published:** 2025-12-05

**Authors:** Carlos Eduardo Poli-de-Figueiredo, Daniele Cristóvão Escouto

**Affiliations:** 1 Pontifícia Universidade Católica do Rio Grande do Sul, Escola de Medicina, Medicina Interna e Nefrologia, Porto Alegre, RS, Brasil.

Pregnancy in women with glomerulopathies remains one of the most complex challenges in nephrology. Beyond the physiological stresses of gestation, both physicians and women face uncertainty at every step, from preconception counseling to the management of maternal risks, fetal outcomes, and long-term renal health. Practicing nephrologists must be prepared not only to manage disease activity during pregnancy but also to support patients in balancing motherhood with the lifelong demands of chronic kidney disease (CKD). Despite decades of research in obstetric nephrology, significant evidence gaps persist regarding glomerulopathies specifically.

Several barriers complicate the diagnosis and treatment of glomerular disease in pregnancy. Renal biopsy during pregnancy, though potentially decisive, is often avoided due to procedural risks. Distinguishing disease activity from superimposed preeclampsia remains notoriously difficult, and the absence of validated pregnancy-specific biomarkers leaves clinicians without reliable tools. Measuring angiogenic placental proteins may be useful to predict and diagnose pregnant women with a suspicion of preeclampsia^1^. The therapeutic armamentarium is equally constrained: only a handful of immunosuppressive agents are considered safe in pregnancy, and one of the most effective treatments for proteinuric glomerulopathies—renin-angiotensin system blockade—is contraindicated. Thus, clinical judgment, close monitoring, and multidisciplinary teamwork continue to compensate for the lack of robust evidence.

A consistent finding in the literature is that renal function at conception is a strong predictor of maternal outcomes^2,3,4^. Proteinuria, chronic hypertension, and preeclampsia are believed to worsen prognosis, but their independent contributions remain difficult to disentangle^2,3,4,5,6^. Historically, most data have come from retrospective cohorts that combined women with diverse etiologies of CKD. Except for lupus nephritis and, to a lesser extent, IgA nephropathy and ANCA vasculitis, disease-specific evidence has been scarce. For common conditions such as membranous nephropathy or focal segmental glomerulosclerosis, counseling has often relied more on clinical intuition than on robust data^7^.

In this context, the recent Brazilian study by Marques et al.^8^, published in the Brazilian Journal of Nephrology, represents a significant step forward. Conducted at a single center in Rio de Janeiro, the study followed 44 planned pregnancies in 38 women with biopsy-proven glomerulopathies, monitored for at least one year postpartum. The findings were sobering: eight women progressed to end-stage kidney disease during pregnancy, most of them with advanced CKD stages at conception. However, the risk was not negligible even at stages I-IIIb, as women with preserved renal function also experienced a measurable decline in glomerular filtration rate postpartum. Preeclampsia emerged as a particularly harmful factor, accelerating kidney function loss. Overall, adverse maternal outcomes occurred in more than half of the pregnancies, while adverse fetal outcomes—including preterm birth, low birth weight, and fetal death—were seen in nearly two-thirds of cases.

What distinguishes this study is not only the detailed, biopsy-confirmed diagnoses, but also its emphasis on long-term maternal outcomes. It highlights that pregnancy’s impact on glomerular disease extends well beyond delivery, influencing future cardiovascular and renal health. Moreover, it demonstrates that meaningful insights can be obtained even from small, well-designed cohorts— provided there is careful planning, multidisciplinary care, and sustained follow-up.

Despite including only planned pregnancies, the study did not clarify whether structured preconception counseling was provided. It is important to highlight the need for pre-pregnancy counseling to empower women and their partners to make a conscious and shared decision. Apart from the use of acetylsalicylic acid to prevent preeclampsia^9^, there are currently few therapeutic options available to reduce adverse outcomes for both the mother and the fetus.

In addition, the study underscores how much remains unknown and how urgently coordinated efforts are needed. Latin America is uniquely positioned to lead this agenda. The region’s diverse patient populations, high prevalence of CKD, and established nephrology networks offer fertile ground for collaborative registries and multicenter research. If a single Brazilian center can generate such valuable data, the potential impact of a regional registry spanning multiple institutions and countries could be transformative.

Pregnancy is possible for women with glomerular disease, but it remains a high-risk endeavor for both mother and child. Planned conception, individualized counseling, and multidisciplinary care improve outcomes, but risks persist. As the Brazilian study reminds us, the consequences of pregnancy extend beyond the delivery room—shaping maternal renal trajectories and influencing the long-term health of infants, many of whom are born preterm or underweight.

We owe it to our patients not only to support them through pregnancy but also to generate data that will make future pregnancies safer, more predictable, and more equitable. Establishing multicenter registries, advancing biomarker discovery, and embedding preconception counseling into routine nephrology practice are urgent next steps for the field.

## Data Availability

No new data were generated or analyzed in this study.
