# Supplementary material for: Glomerular disease and pregnancy: contributions from a Brazilian cohort study
Source: J Bras Nefrol. 2025 Dec 5;47(4):e2025E020. doi: 10.1590/2175-8239-JBN-2025-E020en (PMC12704872; doi:10.1590/2175-8239-JBN-2025-E020en)
Supplement: Translation

# Translation: Doença glomerular e gestação: contribuições de um estudo de coorte brasileiro

**DOI:** 10.1590/2175-8239-JBN-2025-E020pt

**Authors:** Carlos Eduardo Poli-de-Figueiredo, Daniele Cristóvão Escouto

**Affiliations:** 1 Pontifícia Universidade Católica do Rio Grande do Sul, Escola de Medicina, Medicina Interna e Nefrologia, Porto Alegre, RS, Brasil.

A gestação em mulheres com glomerulopatias permanece um dos desafios mais complexos da nefrologia. Além dos estresses fisiológicos da gestação, tanto os médicos quanto as mulheres enfrentam incertezas em cada etapa, desde o aconselhamento pré-concepcional até o manejo dos riscos maternos, desfechos fetais e saúde renal em longo prazo. Os nefrologistas em exercício devem estar preparados não apenas para o manejo da atividade da doença durante a gravidez, mas também para apoiar as pacientes no equilíbrio entre a maternidade e as demandas ao longo da vida, decorrentes da doença renal crônica (DRC). Apesar de décadas de pesquisa em nefrologia obstétrica, ainda existem lacunas de evidências significativas, especificamente no que diz respeito às glomerulopatias.

Diversas barreiras dificultam o diagnóstico e o tratamento da doença glomerular na gestação. Embora potencialmente decisiva, a biópsia renal durante a gravidez é frequentemente evitada devido aos riscos inerentes ao procedimento. Distinguir entre atividade da doença e pré-eclâmpsia sobreposta continua sendo notoriamente complexo, e a ausência de biomarcadores validados específicos para a gestação deixa os médicos sem ferramentas confiáveis. A medição das proteínas angiogênicas placentárias pode ser útil na predição e no diagnóstico de gestantes com suspeita de pré-eclâmpsia^1^. O arsenal terapêutico é igualmente limitado: apenas alguns agentes imunossupressores são considerados seguros durante a gravidez, e um dos tratamentos mais eficazes para glomerulopatias proteinúricas — o bloqueio do sistema renina-angiotensina — é contraindicado. Dessa forma, o julgamento clínico, o monitoramento rigoroso e o trabalho da equipe multidisciplinar continuam a suprir a falta de evidências robustas.

Um achado consistente na literatura é que a função renal no momento da concepção constitui um forte preditor de desfechos maternos^2,3,4^. Acredita-se que a proteinúria, a hipertensão arterial crônica e a pré-eclâmpsia agravem o prognóstico, mas suas contribuições independentes ainda são difíceis de distinguir^2,3,4,5,6^. Historicamente, a maioria dos dados provém de coortes retrospectivas que reuniram mulheres com diferentes etiologias de DRC. Com exceção da nefrite lúpica e, em menor grau, da nefropatia por IgA e das vasculites associadas ao ANCA, as evidências específicas para cada doença têm sido escassas. Para condições comuns, como nefropatia membranosa ou glomeruloesclerose segmentar e focal, o aconselhamento tem, com frequência, se baseado mais na intuição clínica do que em dados robustos^7^.

Nesse contexto, o recente estudo brasileiro de Marques et al.^8^, publicado no *Brazilian Journal of Nephrology*, representa um avanço significativo. Realizado em um único centro no Rio de Janeiro, o estudo acompanhou 44 gestações planejadas em 38 mulheres com glomerulopatias comprovadas por biópsia, monitoradas por pelo menos um ano após o parto. Os achados foram preocupantes: oito mulheres evoluíram para doença renal em estágio terminal durante a gestação, a maioria delas apresentando estágios avançados de DRC na concepção. No entanto, o risco não foi insignificante mesmo nos estágios I-IIIb, uma vez que mulheres com função renal preservada também apresentaram um declínio mensurável na taxa de filtração glomerular no pós-parto. A pré-eclâmpsia surgiu como um fator particularmente prejudicial, acelerando a perda da função renal. No geral, desfechos maternos adversos ocorreram em mais da metade das gestações, enquanto desfechos fetais adversos — incluindo parto prematuro, baixo peso ao nascer e óbito fetal — foram observados em quase dois terços dos casos.

O que distingue este estudo não são apenas os diagnósticos detalhados e confirmados por biópsia, mas também sua ênfase nos desfechos maternos de longo prazo. O trabalho destaca que o impacto da gestação na doença glomerular se estende muito além do parto, influenciando a saúde cardiovascular e a saúde renal futuras. Além disso, demonstra que insights significativos podem ser obtidos mesmo a partir de coortes pequenas e bem desenhadas, desde que haja um planejamento cuidadoso, cuidado multidisciplinar e acompanhamento contínuo.

Apesar de incluir somente gestações planejadas, o estudo não esclareceu se foi oferecido aconselhamento pré-concepcional estruturado. É importante destacar a necessidade desse aconselhamento para empoderar as mulheres e seus parceiros a tomarem uma decisão consciente e compartilhada. Além do uso de ácido acetilsalicílico para a prevenção da pré-eclâmpsia^9^, atualmente existem poucas opções terapêuticas disponíveis para reduzir os desfechos adversos, tanto maternos quanto fetais.

Além disso, o estudo ressalta o quanto ainda não se sabe e a urgência de esforços coordenados. A América Latina ocupa uma posição privilegiada para liderar essa agenda. A diversidade da população de pacientes da região, a alta prevalência de DRC e as redes de nefrologia estabelecidas oferecem um terreno fértil para registros colaborativos e pesquisas multicêntricas. Se um único centro brasileiro pode gerar dados tão valiosos, o impacto potencial de um registro regional abrangendo múltiplas instituições e países poderia ser transformador.

A gestação é possível para mulheres com doenças glomerulares, mas continua sendo um empreendimento de alto risco tanto para a mãe quanto para o filho. A concepção planejada, o aconselhamento individualizado e o cuidado multidisciplinar contribuem para melhores desfechos, porém os riscos permanecem. Como nos lembra o estudo brasileiro, as consequências da gestação ultrapassam a sala de parto — moldando a trajetória renal materna e influenciando a saúde a longo prazo dos bebês, muitos dos quais nascem prematuros ou com baixo peso.

Temos o compromisso com nossas pacientes não apenas de apoiá-las durante a gestação, mas também de gerar dados que tornem as futuras gestações mais seguras, previsíveis e equitativas. Estabelecer registros multicêntricos, avançar na descoberta de biomarcadores e incorporar o aconselhamento pré-concepcional à prática nefrológica de rotina representam os próximos passos de urgência para a área.

## Data Availability

Nenhum dado novo foi gerado ou analisado neste estudo.
